# The fibronectin III-1 domain activates a PI3-Kinase/Akt signaling pathway leading to αvβ5 integrin activation and TRAIL resistance in human lung cancer cells

**DOI:** 10.1186/s12885-016-2621-6

**Published:** 2016-08-02

**Authors:** Christina Cho, Carol Horzempa, David Jones, Paula J. McKeown-Longo

**Affiliations:** 1 Center for Cell Biology & Cancer Research (MC-165), Albany Medical College, 47 New Scotland Avenue, Albany, NY 12208 USA; 2 Department of Pathology and Laboratory Medicine, Albany Medical College, 47 New Scotland Avenue, Albany, NY 12208-3479 USA

**Keywords:** Fibronectin, Akt, Integrin, TRAIL, Vitronectin

## Abstract

**Background:**

Fibronectin is a mechanically sensitive protein which is organized in the extracellular matrix as a network of interacting fibrils. The lung tumor stroma is enriched for fibronectin which is thought to contribute to metastasis and drug resistance. Fibronectin is an elastic, multi-modular protein made up of individually folded domains, some of which can stretch in response to increased mechanical tension. Very little is known about the relationship of fibronectin’s unfolded domains to lung cancer resistance to chemotherapy. In the present study, we evaluated the impact of unfolding the first Type III domain of fibronectin (FnIII-1c) on TNF-related apoptosis inducing ligand (TRAIL) resistance.

**Methods:**

NCI-H460 non-small cell lung cancer cells were treated with FnIII-1c then assessed for TRAIL-induced apoptosis. Subsequent analysis of FnIII-1c-mediated signaling pathways was also completed. Human non-small cell lung cancer tissue sections were assessed for the expression of vitronectin by immunohistochemistry.

**Results:**

FnIII-1c inhibited TRAIL-induced activation of caspase 8 and subsequent apoptosis in NCI-H460 lung cancer cells. FnIII-1c treatment was associated with the activation of the phosphatidylinositol-3-kinase/alpha serine/threonine kinase (PI3K/Akt) pathway and the αvβ5 integrin receptor for vitronectin, both of which were required for TRAIL resistance. Immunohistochemical staining of sections from non-small cell lung cancers showed that vitronectin was localized around blood vessels and in the tumor-stroma interface.

**Conclusions:**

Unfolding of Type III domains within the fibronectin matrix may promote TRAIL resistance through the activation of a PI3K/Akt/αvβ5 signaling axis and point to a novel mechanism by which changes in secondary structure of fibronectin contribute to cancer cell resistance to apoptosis.

## Background

Cancers develop in a mechanically and biologically active microenvironment that continuously evolves with the disease. The tumor microenvironment is desmoplastic – abundant in infiltrating immune cells, tumor-associated fibroblasts and fibrotic extracellular matrix (ECM) proteins – and this “reactive” stroma distinguishes carcinomas from normal tissues. In addition to desmoplasia, the tumor stroma is characterized by deregulated ECM remodeling and tissue stiffening, which are associated with malignant progression [1].

TNF-related apoptosis inducing ligand (TRAIL) is a novel therapeutic agent currently under clinical trial for the treatment of non-small cell lung cancer (NSCLC) [2]. TRAIL binds to death receptors 4 and 5 (DR4, DR5) to induce apoptosis through the extrinsic pathway. Binding of trimeric TRAIL to DR4/5 stimulates receptor oligomerization and the formation of the death inducing signaling complex (DISC). The components of the DISC include Fas-associated protein with death domain (FADD), caspase 8, and cellular FLICE-like inhibitor protein (c-FLIP). Proper formation of the DISC results in the activation and cleavage of caspase 8, which then initiates the apoptotic death program [3]. Preclinical studies implicated TRAIL as an ideal therapy for non-small cell lung cancer (NSCLC). In mouse models of human lung cancer, TRAIL promoted tumor regression, delayed tumor growth, and improved overall survival [4]. In addition, late stage human tumors stained positively for DR4 (99 %) and DR5 (82 %) [5], suggesting that those tumors could be targeted with TRAIL based therapeutics. However, results from clinical trials using DR4 or DR5 agonists in combination with traditional chemotherapies showed no improvement in response rates or progression free survival (PGS) [2]. The failure to translate preclinical success in clinical trials suggests a need for a deeper investigation of the mechanisms regulating death receptor function.

Fibronectin is one of the most common and abundant ECM proteins deposited in the stroma of aggressive tumors [6–8]. In the metastatic niche, fibronectin functions as a scaffold for the continued recruitment of haematopoietic and invading cancer cells [9]. In NSCLC, fibronectin overexpression is associated with increased angiogenesis, enhanced cancer cell survival, and metastasis [10]. Fibronectin is a mechanically sensitive protein whose secondary structure is organized into individually folded domains termed the type I, II and III [11]. Unlike the type I and II domains, fibronectin type III domains lack stabilizing disulfide bonds which allows them to unfold in response to mechanical and cell-contractile forces which are generated in response to increased tissue rigidity [12–15]. Recent studies have shown that tumor-associated fibronectin matrices are stiffer and the fibronectin fibers stretched and unfolded [16]. Very little is known about the impact of these changes in fibronectin secondary structure on either tumor progression or chemoresistance.

Atomic force microscopy and steered molecular dynamics have identified a partially unfolded, stable intermediate of the first type III domain of fibronectin (FnIII-1c) which is predicted to form in response to contractile unfolding [12]. In this study, we investigated the impact of the unfolded FnIII-1 on TRAIL-induced apoptosis in NSCLC cells using the FnIII-1c peptide to recapitulate the unfolded FnIII-1 structure [12]. We found that FnIII-1c inhibited TRAIL-induced apoptosis via a PI3K-Akt dependent activation of integrin αvβ5. Additionally, we detected vitronectin, the ligand for integrin αvβ5, in human NSCLC tumors surrounding blood vessels and in the interstitium between the tumor and stroma. Our data suggest that the changes in fibronectin secondary structure, which occur in response to the increased tissue rigidity of the tumor stroma, may contribute to apoptosis resistance.

## Methods

### Antibodies and reagents

All reagents were purchased from Sigma (St. Louis, MO) unless indicated otherwise. Fetal bovine serum (FBS) was from Hyclone (Logan, UT). Recombinant fibronectin type III domains FnIII-1c, FnIII-10n and FnIII-13 were generated and purified as previously described [17]. Recombinant human TRAIL/TNFSF10 was purchased from R&D systems (Minneapolis, MN). The PI3K inhibitor LY294002 was purchased from Biomol (Plymouth Meeting, PA) and the Akt inhibitor VIII (AG730) was purchased from Sigma. Polyclonal rabbit antibody against phospho-Akt (Ser473), Hoechst 33342 stain for visualization of nuclei, and the Click-iT® TUNEL AlexaFluor® Imaging assay were purchased from Invitrogen (Carlsbad, CA). The rabbit monoclonal antibodies against cleaved caspase 8 and GAPDH were both purchased from Cell Signaling Technology (Danvers, MA). Mouse monoclonal antibodies against integrin αvβ5 (15 F11), integrin β3 (LM609), integrin αv (MAB1980), α5β1, and the monoclonal blocking antibody against integrin αvβ5 (P1F6) were purchased from Millipore (Billerica, MA). The purified rat anti-mouse CD29 (clone 9EG7) which recognizes the ligand bound conformation of the β1 integrin [18] was purchased from BD Biosciences. The rabbit polyclonal antibody to vitronectin, AC7, has been previously described [19]. Pre-immune normal rabbit IgG was used as a control. Alexafluor488-conjugated secondary anti-mouse IgG (H + L) antibody was purchased from Molecular Probes (Eugene, OR). Horseradish peroxidase conjugated secondary antibodies to mouse IgG (H + L) and rabbit IgG (H + L) were purchased from BioRad (Berkeley, CA).

### Cell culture, treatment and lysis

The human tumor cell line NCI-H460 was purchased from the American Type Culture Collection (Manassas, VA). NCI-H460 cells were grown in monolayer culture in complete medium (RPMI 1640 with streptomycin-penicillin and glutamax supplemented with 10 % FBS) at 37 °C in a humidified atmosphere containing 5 % CO_2_. Prior to treatment, cells were serum-starved in RPMI-1640 with 0.1 % BSA for 2 h. For the collection of whole cell lysates, monolayers were washed twice in ice-cold PBS and lysed in whole cell lysis buffer (100 mM Tris–HCl, pH  6.8, 2 % SDS, 10 % glycerol, 100 mM DTT).

### Terminal dexoynucleotidyl transferase-mediated dUTP nick end labeling (TUNEL) assay for apoptosis

NCI-H460 cells were cultured in complete medium 48 h or until cells reached ~85 % confluency, rinsed once with PBS and serum-starved in RPMI-1640 with 1 % BSA for 2 h at 37 °C in a humidified atmosphere containing 5 % CO_2_. Cells were stimulated with recombinant human TRAIL/TNFSF10 (TRAIL) (R&D systems) or PBS as a control treatment as indicated in the figure legends. Apoptosis was assessed by cleaved caspase 8 protein levels via western blot analysis or by TUNEL assay. The TUNEL assay was performed using Click-It® TUNEL AlexaFluor488® Imaging Kit (Invitrogen) according to the manufacturer’s protocol. In brief, cells were fixed with 4 % paraformaldehyde in PBS at room temperature for 20 min and permeabilized with Triton X-100 (0.25 % in PBS) for an additional 15 min. The cells were then washed twice and incubated with 50 μL of terminal deoxynucleotidyl transferase reaction buffer (Component A) for 10 min at room temperature. The buffer was removed and the cells were incubated with TUNEL reaction mixture containing terminal deoxynucleotidyl transferase for 1 h in a humidified chamber at 37 °C for 1 h. Post treatment, cells were washed three times with 3 % BSA in PBS for 2 min each and then incubated with 50 μL of Click-iT reaction mixture (containing Alexa 488) for 30 min at room temperature, protected from light. The cells were then washed with 3%BSA in PBS and the nuclei were stained with Hoechst 33342 for 1 min at room temperature, protected from light. The coverslips were washed twice with PBS before mounting onto a slide with ProLong® Gold Antifade Mount (Life technologies). Cell monolayers were examined using an Olympus BMX-60 microscope equipped with a cooled CCD sensi-camera (Cooke, Auburn Hills, MI), and images were acquired using Slidebook software (Intelligent Imaging Innovation, Denver, CO). Fluorescence images were processed with ImageJ analysis software for the quantification of TUNEL-positive nuclei and total number of nuclei. The number of TUNEL-positive cells in three random (20×) fields was counted and divided by the total number of nuclei to determine the percentage of TUNEL-positive nuclei.

### Western blot analysis

Whole cell lysates were collected from treated cells and subjected to SDS-PAGE under reducing conditions and transferred onto nitrocellulose membranes (Schleicher and Schuell Bioscience, Keene, NH). Membranes were blocked for either 2 h at room temperature or overnight at 4 °C with 5 % BSA (w/v) in Tris-buffered saline containing 0.1 % Tween-20 then incubated with primary antibodies for 2 h at room temperature or overnight at 4 °C. Blots were washed with Tween-20 and incubated with horseradish peroxidase-linked secondary antibodies (1:10,000) for 1 h at room temperature. Immunoreactive bands were detected using Clarity^TM^ Western ECL substrate (BioRad). Blots were reprobed after stripping in 62.5 mM Tris–HCl, pH 6.8, 2 % SDS and 10 mM β-mercaptoethanol for 20 min at 60 °C. Western blots were quantified either by ImageJ analysis software (for blots developed on film) or by ChemiDoc™ MP Imaging System with Image Lab (BioRad).

### Adhesion assay for integrin activation

Polystyrene non-tissue culture-treated 48-well plates (Greiner bio-one; Monroe, NC) were coated with vitronectin in PBS overnight at 4 °C. Wells were blocked with 3 % BSA in PBS for 2 h in room temperature. Adherent H460 cells were lifted with Cellstripper® (Cellgro) and resuspended in RPMI-1640 containing 0.1 % BSA. Suspended cells (~10^5^) were serum-starved for 1 h then treated with FnIII-1c or PBS, as a control, for an hour at 37 °C. When pharmacological inhibitors to PI3K (Wortmannin, LY294002) and Akt1/2 (VIII) were used, cells were pre-treated with inhibitors for 30 min prior to treatment with FnIII-1c for an additional hour. Treated cells were seeded onto vitronectin-coated wells at a density of approximately 4x10^4^cells/wells and allowed to adhere for 1 h at 37 °C. Adherent cells were quantified by staining with 0.05 % toluidine blue for 1 h. Each well was washed four times and dye was extracted with 10 % acetic acid. Absorbance was measured at 650 nm and corrected for light scattering by subtracting the absorbance at 405 nm.

### Fluorescence microscopy for integrin expression

NCI-H460 cells (~10^4^ cells/mL) were cultured in complete medium for 48 h on glass coverslips, then serum-starved for 1 h. Serum-starved cells were washed once with PBS, fixed for 20 min in 4 % paraformaldehyde, permeabilized in 0.5 % TritonX-100 for 10 min, blocked in 1 % BSA and immunostained with monoclonal antibodies against integrins α5β1 (1:200 dilution), αvβ5 (1:200) αvβ3 (1:100) and 9EG7, a monoclonal antibody that detects ligated β1 integrins (1:100) for 1 h at room temperature or overnight at 4 °C. Slides were then incubated with Alexafluor488-conjugated secondary anti-mouse IgG (H + L) antibody for an additional hour at room temperature. Nuclei were visualized with Hoechst 33342 dye. After staining, slides were mounted with Prolong Antifade according to the manufacturer’s instructions (Molecular Probes) and examined using an Olympus BMX-60 microscope equipped with a cooled CCD sensi-camera (Cooke, Auburn Hills, MI). Images were acquired using Slidebook software (Intelligent Imaging Innovation, Denver, CO).

### Tissue section staining

Non-small cell human lung carcinoma tissue LC241 NSCLC microarray panels (US Biomax Inc., Rockville, MD), were immunostained using the peroxidase-based ABC system (Vector Laboratories, Burlingame, CA). Vitronectin was detected using a polyclonal antibody to vitronectin and for negative controls, the primary antibody was replaced with pre-immune normal rabbit IgG. Color was developed by reaction with 3,3’-Diaminobenzidine. Tissue sections were counterstained with hematoxylin.

### Statistical analysis

Data are presented as the mean ± SE of at least three independent experiments. Adhesion and TUNEL assay results were analyzed using either a one-way or two-way Anova with Tukey’s post-hoc analysis. Statistical analysis was performed with GraphPad Prism 6 with *p* < 0.05 considered significant.

## Results

### FnIII-1c desensitizes NCI-H460 cells to TRAIL-induced apoptosis

**Fig. 1:**
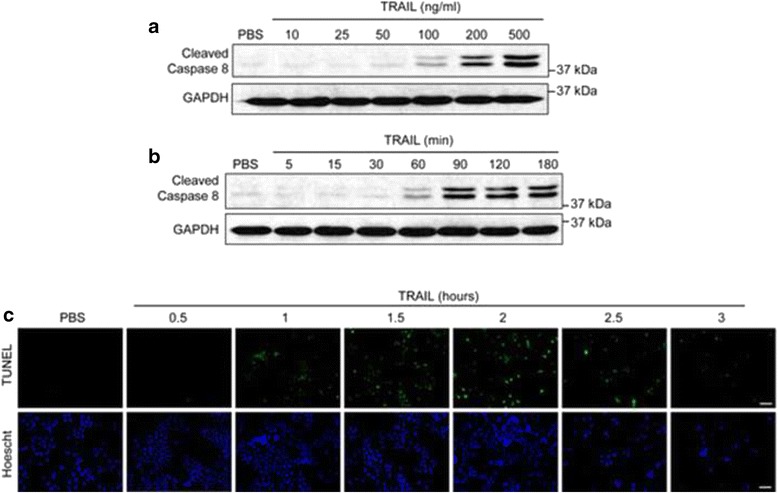
NCI-H460 cells are sensitive to TRAIL-induced apoptosis. Whole cell lysates were collected from NCI-H460 cells treated with various concentrations of TRAIL for 1 h (**a**) or 100 ng/ml of TRAIL for the indicated time (**b**). Lysates were subjected to western blot analysis for cleaved caspase 8 then stripped and reprobed for GAPDH as a loading control. **c** NCI-H460 cells were treated with 50 ng/ml TRAIL for the indicated times and apoptosis was assessed by TUNEL assay. Cells were counterstained with Hoechst 33342 dye to denote nuclei. PBS served as control. Bar = 50 μm

The NCI-H460 cell line was used to evaluate the effect of the unfolded fibronectin type III-1 domain on TRAIL-induced apoptosis. To determine the sensitivity of NCI-H460 cells to TRAIL we performed dose and kinetics studies using recombinant human TRAIL and evaluated two indicators of apoptosis: caspase 8 cleavage and TUNEL Assay. The TUNEL assay detects the DNA fragmentation which occurs in cells undergoing apoptosis. The binding of TRAIL to DR4/DR5 stimulates receptor aggregation and recruitment of caspase 8 to the receptor cytoplasmic domain resulting in the cleavage of caspase 8 and the initiation of the apoptotic program. Cell monolayers were treated with increasing concentrations of TRAIL for 1 h and assessed for cleaved caspase 8 by western blotting. Caspase 8 cleavage was detected at 1 h using 100 ng/ml TRAIL with higher amounts seen in cells treated with 200 and 500 ng/ml TRAIL (Fig. 1a). Kinetic studies using a fixed amount of TRAIL (100 ng/ml) indicated that caspase 8 cleavage occurred after 60 min reaching maximal levels within 90 min of treatment (Fig. 1b). Similar observations were made with the apoptosis assay, in which TUNEL staining was seen within 1 h following treatment with 50 ng/ml TRAIL (Fig. 1c). As evident from the nuclear staining (Hoechst), TRAIL treatment also resulted in the loss of apoptotic cells from the substrate.

**Fig. 2:**
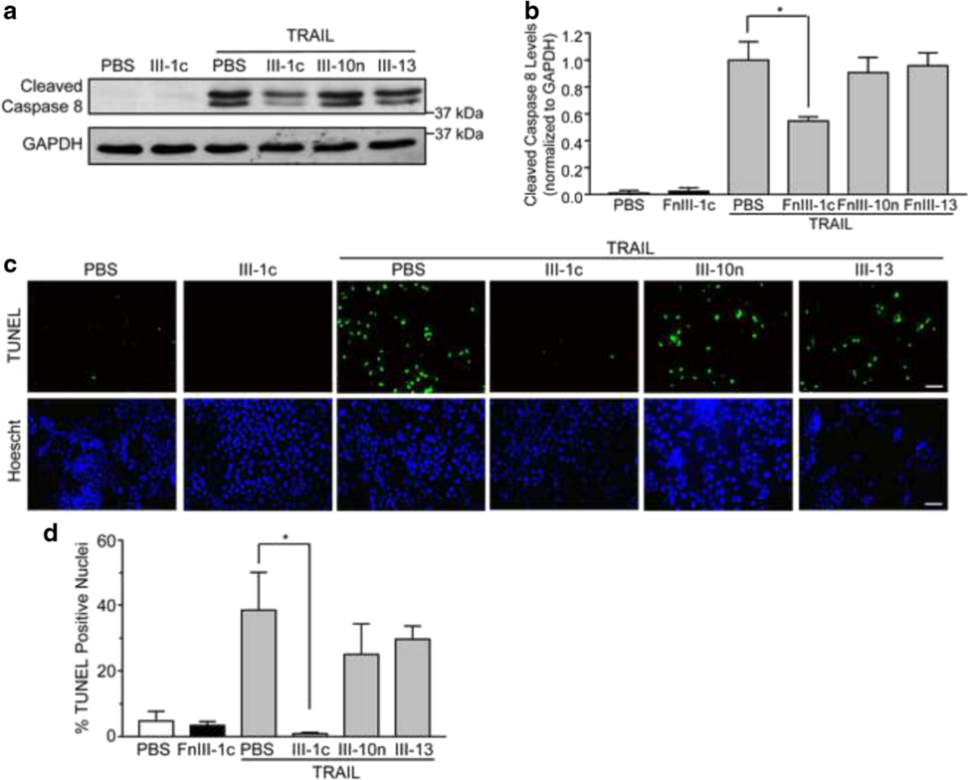
FnIII-1c desensitizes NCI-H460 cells to TRAIL-induced apoptosis. NCI-H460 cells were pre-treated with 10 μM FnIII-1c, FnIII-10n, FnIII-13, or PBS for 1 h, then stimulated with 50 ng/ml TRAIL for an additional 2.5 h. **a** Cell lysates were analyzed by western blotting for expression of cleaved caspase 8 and GAPDH. **b** Densitometric quantification of the data shown in (**a**). Corresponding densitometry for cleaved caspase 8 presented as a change in protein expression relative to TRAIL-treated cells. Each bar represents the mean ± SE of three independent experiments. A one-way ANOVA with Tukey post-hoc analysis was used to determine statistical significance (*p < 0.05). **c** Apoptotic Cells were visualized by TUNEL assay and stained with Hoescht 33342 dye to denote nuclei. Bar = 50 μm. **d** The percentage of TUNEL positive cells was determined by Image J analysis. Each bar represents the mean ± SE of five independent experiments. A one-way Anova with Tukey post-hoc analysis was used to determine statistical significance (*, *p* < 0.05)

To examine the effect of fibronectin Type III domain unfolding on TRAIL signaling, NCI-H460 cells were pre-treated with the FnIII-1c peptide for 1 h, and then incubated with TRAIL for an additional 2.5 h. Whole cell lysates were collected and analyzed for cleaved caspase 8 by western blotting. NCI-H460 cells treated with TRAIL alone expressed cleaved caspase 8 (Fig. 2a), while control cells treated with PBS or FnIII-1c did not. Pre-treatment of cells with FnIII-1c significantly decreased the amount of caspase 8 cleavage in response to TRAIL (Fig. 2a–b). This inhibition was specific to FnIII-1c, as pre-treatment with other fibronectin type III domains (FnIII-10n and FnIII-13) had no significant effect on TRAIL-mediated caspase 8 cleavage (Fig. 2a–b). FnIII-10n and FnIII-13 were selected as controls due to shared characteristics with the FnIII-1c domain, i.e., heparin binding activity (FnIII-13) [20], mechanically unfolded stable intermediate structure (FnIII-10n) [6]. We also examined the amount of TUNEL staining in cells treated with TRAIL in the absence or presence of FnIII-1c (Fig. 2c–d). As shown in panel C, pre-incubation of cells with FnIII-1c greatly decreased TUNEL staining in TRAIL treated cells (Fig. 2c–d). Pretreatment of cells with control type III domains, III-10n and III-13, had no effect on TUNEL staining in response to TRAIL (Fig. 2c–d).

### FnIII-1c enhances the PI3K-Akt-dependent activation of the αvβ5 integrin

**Fig. 3:**
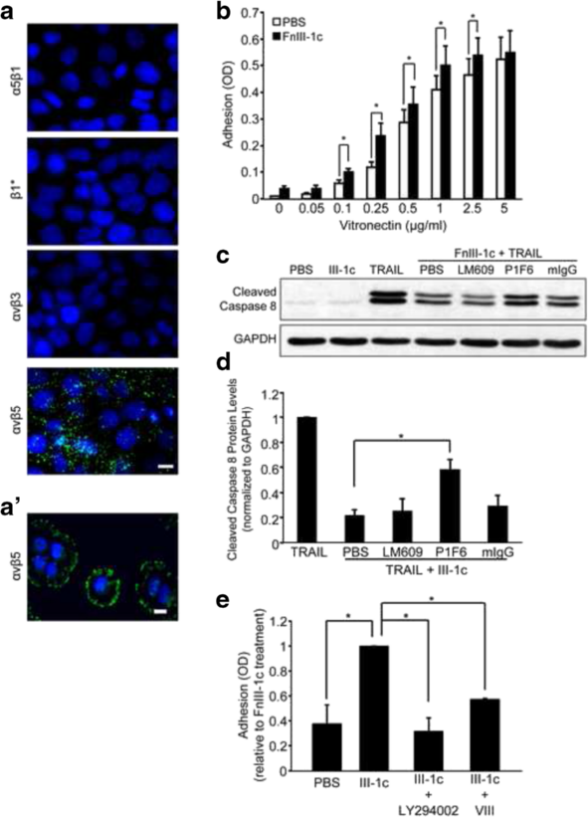
FnIII-1c stimulates the PI3K-Akt-dependent activation of the αvβ5 integrin. **a** NCI-H460 cells were seeded in complete medium for 48 h and then immunostained (FITC) for the indicated integrins. Positive staining was seen only for αvβ5 integrin. Panel A' shows αvβ5 integrins in focal contacts of individual cells. Bar = 10 μm. Nuclei were counterstained with Hoechst 33342. **b** NCI-H460 cells were pretreated with 10 μM FnIII-1c or PBS for 1 h before seeding onto wells coated with the designated concentrations of vitronectin. After 1 h, cell adhesion was measured by toluidine staining. The data are presented as the mean absorbance (OD) ± SE of at least 3 independent experiments. Results were analyzed by a two-way Anova with Tukey’s post-hoc analysis (*, *p* < 0.05). **c** H460 cell monolayers were incubated with blocking antibody to the αvβ3 (LM609), αvβ5 (P1F6) integrin or control mouse IgG. Cells were then treated with 10 μM FnIII-1c for 1 h prior to stimulation with 100 ng/ml TRAIL for an additional 2.5 h. Lysates were immunoblotted for cleaved caspase 8 and GAPDH. Cells treated with PBS or FnIII-1c served as additional controls. A representative blot is shown. **d** Densitometric quantification of the data shown in (**c**). Data are presented as the amount of cleaved caspase 8 relative to TRAIL-treated cells, which was set at 1. Each bar represents the mean ± SE of three independent experiments. A one-way ANOVA with Tukey post-hoc analysis was used to determine statistical significance (*, *p* < 0.05). **e** NCI-H460 cells were pre-treated with 10 μM VIII (Akt1/2 kinase inhibitor) or 10 μM LY294002 (PI3K inhibitor) for 30 min before treatment with FnIII-1c. Cells treated with PBS served as control. Treated cells were seeded onto plates coated with vitronectin (0.5 μg/ml) and allowed to adhere for 1 h. Adhesion was measured by toluidine staining (OD) and presented as fold-change relative to FnIII-1c-treated cells, which was set at 1. The data represent the mean ± SE of three independent experiments. Results were analyzed by a two-way Anova with Sidak’s multiple comparison tests (*, *p* < 0.05)

Integrins are a family of cell adhesion receptors known to be involved in tumor progression [21] and aberrant integrin activation is associated with increased metastasis, enhanced cancer cell survival, and resistance to chemotherapy [22]. Earlier studies have shown that integrins negatively regulate Fas induced apoptosis [23]; therefore, we investigated whether integrins were involved in FnIII-1c-mediated inhibition of TRAIL-induced apoptosis. To identify the integrins mediating adhesion of NCI-H460 cells to the substrate, cells cultured for 48 h were stained with monoclonal antibodies to integrins α5β1, αvβ5, αvβ3, and also with a β1 antibody (9EG7) directed against ligand-occupied β1 integrins (β1*) [18]. Only the vitronectin binding integrin, αvβ5, was localized in clusters typical of adhesion complexes (Fig. 3a). In agreement with earlier studies showing low levels of αvβ3 on NCI-H460 cells [24], there was no staining for integrin αvβ3. There was also no staining for the α5β1 integrin consistent with earlier reports showing low levels of fibronectin synthesis by NCI-H460 cells [25]. Staining with the 9EG7 antibody was also negative. Both the αvβ3 and β1 integrins could be stained in control experiments using fibroblasts (data not shown). These results indicate that adhesion of H460 cells was dependent on the interaction of integrin αvβ5 with vitronectin deposited on the substrate from the serum (Fig. 3a).

To test whether FnIII-1c was affecting the activation state of integrin αvβ5, we evaluated cell adhesion over a range of vitronectin concentrations. FnIII-1c or PBS control treated cells were seeded onto vitronectin-coated plates and allowed to adhere for 1 h. As displayed in panel B, FnIII-1c significantly increased cell adhesion to vitronectin across a range of coating concentrations, consistent with an increase in integrin activation (Fig. 3b). Experiments were then done to determine whether the change in integrin activation might be required for FnIII-1c’s inhibition of TRAIL-induced apoptosis. Treatment of cells with a blocking antibody to αvβ5 (P1F6) partially restored TRAIL-induced caspase 8 cleavage in FnIII-1c treated cells (Fig. 3c). Analysis of data from several experiments indicated that this restoration was statistically significant (Fig. 3d). Control experiments using LM609, the integrin αvβ3 blocking antibody, or a non-immune mouse IgG, had no effect on FnIII-1c-mediated inhibition of TRAIL-induced caspase 8 cleavage (Fig. 3c–d). These data suggest that the increase in activation and ligation of integrin αvβ5 was contributing to FnIII-1c-mediated inhibition of TRAIL-induced apoptosis.

**Fig. 4:**
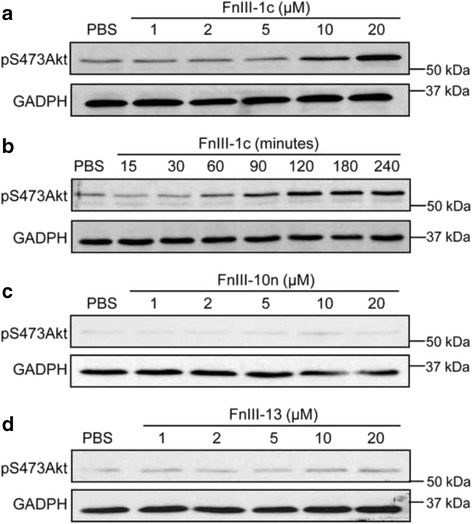
FnIII-1c induces Akt phosphorylation in NCI-H460 cells. NCI-H460 cells were treated with various concentrations of FnIII-1c for 1 h (**a**) or with 10 μM FnIII-1c for increasing lengths of time (**b**). NCI-H460 cells were treated with the control modules, FnIII-10n (**c**) or FnIII-13 (**d**), at the designated concentrations for 1 h. PBS served as control. Cell lysates were analyzed by western blotting for phosphorylated Akt (pS473Akt). GAPDH served as loading control

Previous studies have shown that the PI3K-Akt pathway can regulate the activation of integrin receptors through inside-out signaling [26]; therefore, we investigated whether Akt was involved in FnIII-1c-mediated activation of integrin αvβ5. Two inhibitors of the PI3K-Akt pathway, VIII and LY294002 (Akt1/2 inhibitor [27] and PI3K inhibitor, respectively) were tested for their effects on FnIII-1c-mediated activation of the αvβ5 integrin. As shown in Fig. 3e, both LY294002 and VIII significantly attenuated FnIII-1c’s ability to increase adhesion to vitronectin. A similar result was obtained using the PI3K inhibitor, Wortmannin (data not shown). These data suggest that FnIII-1c-mediated activation of integrin αvβ5 is dependent on the PI3K-Akt pathway. To evaluate the effect of FnIII-1c on Akt activation, NCI-H460 cells were treated with various concentrations of FnIII-1c for 1 h and the activation of Akt, as indicated by the phosphorylation of Akt at serine 473 (pS473Akt), was assessed by western blotting. Akt phosphorylation in response to FnIII-1c was observed at 5–10 μM (Fig. 4a) within 1 h and remained elevated for several hours (Fig. 4b). The activation of Akt was specific to FnIII-1c, as treatment with the control modules, FnIII-10n (Fig. 4c) and FnIII-13 (Fig. 4d), did not result in an increase in pS743Akt.

### Inhibition of the PI3K-Akt pathway restores TRAIL-induced apoptosis in FnIII-1c-treated NCI-H460 cells

**Fig. 5:**
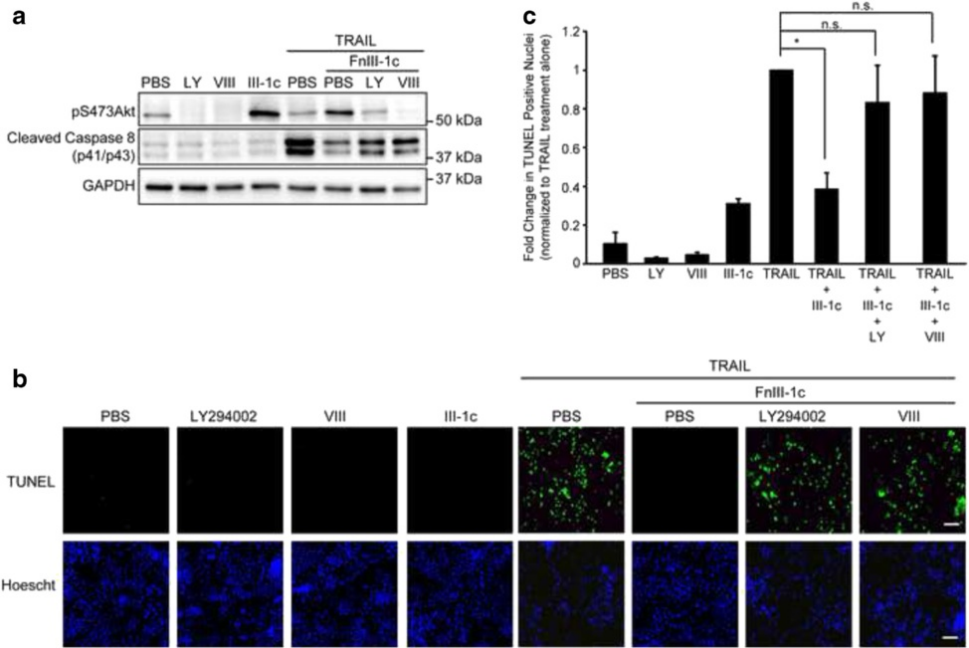
Inhibition of the PI3K-Akt pathway restores TRAIL-induced apoptosis in FnIII-1c treated NCI-H460 cells. NCI-H460 cells were pre-incubated with the Akt inhibitor VIII (10 μM) or the PI3 kinase inhibitor LY294002 (10 μM) for 1 h. FnIII-1c (20 μM) was added for an additional hour and cells were stimulated with 100 ng/ml TRAIL for 2.5 h. PBS served as control. **a** Whole cell lysates were collected and subjected to western blot analysis for phosphorylated Akt (pS473Akt) and cleaved caspase 8. GAPDH was used as a loading control (**b**). Alternatively cells were evaluated for apoptosis by TUNEL assay and stained with Hoechst 33342 dye to visualize nuclei. Bar - 50 μm. **c** Quantification of the data shown in (**b**). TUNEL positive nuclei were determined in three independent experiments using Image J analysis. TUNEL positive nuclei were determined using Image J. Statistical analysis was done using a One-Way Anova with Tukey post-hoc analysis to determine significance. (*, *p* < 0.01; n.s = not significant)

In order to evaluate the role of Akt in regulating the inhibition of TRAIL signaling by FnIII-1c, NCI-H460 cells were pre-treated with PI3K and Akt inhibitors prior to incubation with FnIII-1c and TRAIL and assessed for Akt activation and caspase 8 cleavage by western blot. Treatment of cells with PI3K and Akt inhibitors alone reduced baseline levels of pS473Akt, but did not induce caspase 8 cleavage (Fig. 5a) or TUNEL staining (Fig. 5b, c) suggesting that inhibition of the PI3K-Akt pathway alone was not sufficient to initiate apoptotic pathways. TRAIL treatment resulted in caspase 8 cleavage, which was attenuated by the addition of FnIII-1c (Fig. 5a). Both inhibitors of the PI3K-Akt pathway, VII and LY294002, restored TRAIL-dependent caspase 8 cleavage, indicating that FnIII-1c-mediated inhibition of TRAIL-signaling is dependent on the PI3K-Akt pathway. Similar results were seen using TUNEL assays. As shown in Fig. 5b and quantitated in Fig. 5c, incubation of cells with TRAIL resulted in an increase in TUNEL staining, which was completely prevented by pretreatment with FnIII-1c. Pre-incubation of cells with the inhibitors of the PI3-Akt pathway, VIII and LY294002, restored TRAIL-induced apoptosis in the presence of FnIII-1c. These data were quantified across three independent experiments (Fig. 5c). Taken together, these data suggest that the unfolded III-1 domain of fibronectin mediates TRAIL resistance of NCI-H460 cells by activation of the αvβ5 integrin through the PI3K/Akt signaling pathway.

### Human NSCLC tumors express vitronectin

**Fig. 6:**
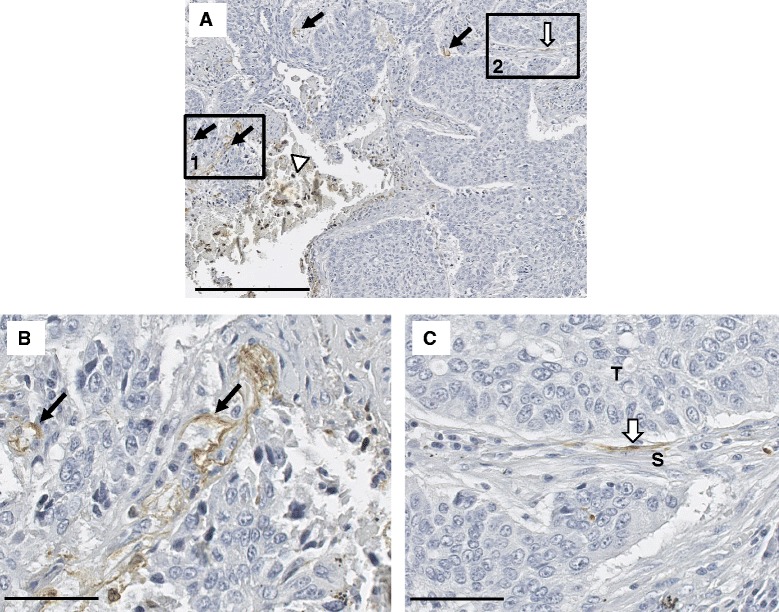
Immunohistochemical localization of vitronectin in human NSCLC tumors. (**a**) Low-magnification image of a representative tumor section of squamous cell carcinoma. Vitronectin expression is seen surrounding blood vessels (*black arrows, box 1*), in the tumor-stroma interface (*open arrow, box 2*), and associated with necrotic tissue (*open arrowhead*). Scale bar = 250 μm. (**b**) High-magnification image of box 1 in panel **a**. Vitronectin positive staining in a collapsed small artery (*arrow on right*) and an arteriole (*arrow on left*) located in the stroma. (**c**) Highmagnification image of box 2 in panel **a**. Vitronectin-positive staining in the interstitial space between tumor cells (T) and adjacent normal stroma (S). Scale bar in (**b**) and (**c**) = 50 μm

Our data suggest that unfolding of Type III domains within the fibronectin matrix may promote TRAIL resistance which depends on increased activation and ligation of αvβ5 integrin receptors. The primary ligand for the αvβ5 integrin is the plasma protein vitronectin [28]. High levels of ECM proteins are found in the NSCLC stroma [29] and the overexpression of fibronectin has been well documented to be associated with metastatic progression and drug resistance [30]. Much less is known about the distribution and function of vitronectin in NSCLC. Using a tissue array, we examined the localization of vitronectin in human NSCLC lung tumor sections. NSCLC human tumor samples were immunostained for vitronectin (Fig. 6a). VN expression was observed (Fig. 6a) surrounding blood vessels (filled arrows), in the interstitial space between the stroma and tumor (open arrows), and in areas of necrosis (arrow heads). In addition to being quite prominent in areas of necrosis, vitronectin staining was evident consistently around the arterioles (Fig. 6b, black arrow on left) and small arteries (Fig. 6b, black arrow on right). Positive staining was also seen in the tumor-stromal interface. This staining was not present throughout the entire interface but was restricted to small localized areas (Fig. 6c). The source of the vitronectin in these tissues is not known but may arise from the leakage of plasma proteins around blood vessels and localized synthesis by cells within the stroma. Although vitronectin is not typically synthesized by cells other than hepatocytes, reports have documented vitronectin synthesis by monocytes/macrophages [31] and lung epithelial cells [32]. Taken together, the data are consistent with a model whereby tumor cells proximal to both fibronectin and vitronectin within the stromal matrix represent a cohort of TRAIL-resistant cells.

## Discussion

Changes in tissue mechanical properties is a hallmark of solid tumors. Lung cancer is often seen in association with pulmonary diseases characterized by increased tissue rigidity secondary to fibrosis, inflammation and extracellular matrix remodeling. Fibronectin is under complex mechanical regulation and the impact of this regulation on progression of solid tumors is not well understood (reviewed in [33]). In the present study, we define a molecular mechanism by which unfolding of the first Type III domain of fibronectin may protect NSCLC cells from TRAIL-induced apoptosis. The first Type III domain of fibronectin has been shown to unfold in vitro to support fibronectin polymerization [17, 34, 35] and in vivo to regulate skeletal muscle contraction [36]. Treatment of NCI-H460 cells with the FnIII-1c peptide derived from the first type III domain of fibronectin resulted in the inhibition of TRAIL-induced apoptosis. Pre-incubation of FnIII-1c treated cells with PI3K or Akt inhibitors was sufficient to restore TRAIL-induced cell death, indicating that the PI3K-Akt pathway was required for FnIII-1c-mediated inhibition of TRAIL-induced apoptosis. We also found that FnIII-1c caused Akt dependent activation of the αvβ5 integrin which was required for FnIII-1c’s inhibition of TRAIL signaling. Consistent with this finding an earlier study in the TRAIL-resistant NSCLC cell line, A549, reported that compared to wildtype TRAIL, RGD-TRAIL was more cytotoxic. The investigators concluded that the tumoricidal effect of RGD-TRAIL was due to the interaction of the RGD sequence with integrins αvβ3 and αvβ5 [37]. Altogether the data demonstrate that the signaling pathways activated in response to fibronectin Type III domain unfolding may contribute to Trail-resistance.

The stromal matrix of solid tumors is in a constant state of remodeling where changes in the balance of mechanical forces can alter the topographical display of bioactive sites [38, 39]. The Type III domains of fibronectin are mechanically labile and have been shown to unfold in response to increased cellular contractile forces generated in rigid tissues [40, 41]. As tumor tissue is known to be more rigid than the neighboring normal tissue, tumor stroma should be enriched in unfolded Type III domains. In agreement with this, recent studies have identified unfolded Type III domains in the stromal fibronectin present in breast tumors [42, 43], where the subsequent change in topography of the fibronectin matrix causes an integrin “switch” to promote angiogenesis [16].

The mechanisms by which Akt protects cancer cells from apoptosis are varied. In the context of TRAIL signaling, Akt has been shown to inhibit cell death by upregulating c-FLIP expression which competes with caspase 8 for recruitment to FADD thereby preventing proper DISC formation [44, 45]. In our study, FnIII-1c had no effect on cFLIP levels (unpublished observations). Instead FnIII-1c stimulated Akt activation and enhanced cell adhesion to vitronectin. This increased adhesion was attenuated by PI3K/Akt inhibitors consistent with FnIII-1c inducing an Akt mediated “inside-out” activation of the αvβ5 integrin. Earlier studies have documented Akt regulation of the activation state of the α5β1, αvβ3 and αIIβ3 integrins [46–48]. Our study is the first to link Akt to αvβ5 integrin activation and suggests that control of integrin activation by Akt may be context dependent. Integrin binding to extracellular matrix is known to promote survival and protect tumor cells against cell death. In many instances, the FAK/Src/PI3K/Akt signaling axis activated by integrin ligation inhibits apoptosis by regulating the expression of anti-apoptotic proteins and cell cycle regulatory genes to prevent both intrinsic and extrinsic cell death (reviewed in [49]). Very recent studies have shown that fibronectin can overcome the effects of several chemotoxic drugs by mechanisms linked to the activation of Akt [46, 50–53]. In our study, Akt activation preceded integrin activation and prevented the cleavage of caspase 8 consistent with FnIII-1c inhibiting TRAIL signaling by blocking the recruitment of pro-caspase 8 to the DISC.

In addition to their role in the regulation of cell death pathways, caspases can participate in a number of other cellular processes including inflammation, differentiation, proliferation and migration [54]. How these various functional activities of caspases are regulated is not well understood. Caspase 8 is subject to various post-translational modifications such as serine/threonine and tyrosine phosphorylation, ubiquitination and nitrosylation (reviewed in [55]). One of the non-apoptotic functions of caspase 8 is to promote cell migration by interacting with pathways controlling focal adhesion turnover [56]. Association of caspase 8 with focal adhesion proteins is dependent on cell adhesion [57], suggesting that increasing the number of ligated integrins may direct caspase 8 to focal adhesions. Therefore, changes in the pattern of ligated integrins may redirect subcellular localization of caspase 8 resulting in the inability of activated death receptors to recruit a critical mass of procaspase to the developing DISC.

In the present study, we localized vitronectin staining in NSCLC tumors to the stroma surrounding blood vessels and to restricted areas of the tumor-stromal interface. These findings are in agreement with a recent study evaluating the expression of vitronectin and the αvβ5 integrin in 215 primary tumors from NSCLC patients. In this study, 70 % of the tumors stained positively for αvβ5 and vitronectin. αvβ5 was localized on the membrane of tumor cells while vitronectin was seen exclusively in the stroma surrounding the blood vessels [58]. In earlier studies, we have shown that the lung stroma adjacent to the border of the infiltrating tumor is heavily stained for fibronectin and smooth muscle actin suggesting that the fibroblasts aligned along the fibronectin matrix are myofibroblasts [59].

## Conclusion

Taken together, these data identify a novel pathway by which changes in the mechanical forces within the stroma can alter the topography of the fibronectin matrix thereby contributing to cancer cell resistance to therapy-mediated cell death. The stretching of fibronectin coupled with the availability of vitronectin in the tumor stroma creates a specialized niche to protect tumor cells from therapies designed to activate extrinsic cell death pathways. Therefore, coupling therapies directed at TRAIL pathways with those controlling mechanical signaling may provide novel approaches for the treatment of TRAIL resistant tumors.

## Abbreviations

Akt, alpha serine/threonine kinase; DISC, death inducing signaling complex; DR4, death receptor 4; DR5, death receptor 5; ECM, extracellular matrix; FADD, Fas-associated protein with death domain; FLICE, FADD-like interleukin 1 beta concerning enzyme; FnIII fibronectin Type III domain; Fn, fibronectin; NSCLC, non-small cell lung cancer; PGS, progressive free survival; PI3K, phosphatidylinositol-3-kinase; TNF, tumor necrosis factor; TRAIL, tumor necrosis factor related apoptosis inducing ligand

## References

[1] Kharaishvili G, Simkova D, Bouchalova K, Gachechiladze M, Narsia N, Bouchal J (2014). The role of cancer-associated fibroblasts, solid stress and other microenvironmental factors in tumor progression and therapy resistance. Cancer Cell Int.

[2] von Pawel J, Harveyt JH, Spigel DR, Dediu M, Reck M, Cebotaru CL, Humphreys RC, Gribbin JJ, Fox NL, Camidge DR (2014). Phase II trial of mapatumumab, a fully human agonist monoclonal antibody to tumor necrosis factor-related apoptosis-inducing ligand receptor I (TRAIL-R1), in combination with iipaclitaxel and carboplatin in patients with advanced non-small-cell lung cancer. Clin Lung Cancer..

[3] Mellier G, Huang S, Shenoy K, Pervaiz S (2010). TRAILing death in cancer. Mol Aspects Med..

[4] Jin H, Yang R, Ross J, Fong S, Carano R, Totpal K, Lawrence D, Zheng Z, Koeppen H, Stern H (2008). Cooperation of the agonistic DR5 antibody apomab with chemotherapy to inhibit orthotopic lung tumor growth and improve survival. Clin Cancer Res..

[5] Spierings DCJ, de Vries EGE, Timens W, Groen HJM, Boezen HM, de Jong S (2003). Expression of TRAIL and TRAIL death receptors in Stage III non-small cell lung cancer tumors. Clin Cancer Res..

[6] Gee EP, Ingber DE, Stultz CM (2008). Fibronectin unfolding revisited: modeling cell traction-mediated unfolding of the tenth type-III repeat. PLoS ONE..

[7] Zerlaught G, Wolf G (1984). Plasma fibronectin as a marker for cancer and other diseases. Am J Med..

[8] Nagai H, Isemura M, Arai H, Abe T, Shimoda S, Motomiya M, Sato H, Hashimoto K, Takusagawa K, Konno K (1986). Pattern of fibronectin distribution in human lung cancer. J Cancer Res Clin Oncol..

[9] Kaplan RN, Riba RD, Zacharoulis S, Bramley AH, Vincent L, Costa C, MacDonald DD, Jin DK, Shido K, Kerns SA (2005). VEGFR1-positive haematopoietic bone marrow progenitors initiate the pre-metastatic niche. Nature..

[10] Shuman Moss LA, Stetler-Stevenson WG (2013). Influence of stromal components on lung cancer carcinogenesis. J Carcinogene Mutagene.

[11] Oberhauser AF, Badilla-Fernandez C, Carrion-Vazquez M, Fernandez JM (2002). The mechanical hierarchies of fibronectin observed with single-molecule AFM. J Mol Biol..

[12] Gao M, Craig D, Lequin O, Campbell ID, Vogel V, Schulten K (2003). Structure and functional significance of mechanically unfolded fibronectin type III1 intermediates. Proc Natl Acad Sci..

[13] Antia M, Baneyx G, Kubow KE, Vogel V (2008). Fibronectin in aging extracellular matrix fibrils is progressively unfolded by cells and elicits an enhanced rigidity response. Farady Discuss..

[14] Baneyx G, Baugh L, Vogel V (2002). Fibronectin extension and unfolding within cell matrix fibrils controlled by cytoskeletal tension. Proc Natl Acad Sci USA.

[15] Smith ML, Gourdon D, Little WC, Kubow KE, Eguiluz RA, Luna-Morris S, Vogel V (2007). Force-induced unfolding of fibronectin in the extracellular matrix of living cells. PLoS Biol.

[16] Wang K, Andresen Eguiluz RC, Wu F, Seo BR, Fischbach C, Gourdon D (2015). Stiffening and unfolding of early deposited-fibronectin increase proangiogenic factor secretion by breast cancer-associated stromal cells. Biomaterials..

[17] Hocking DC, Sottile J, McKeown-Longo PJ (1994). Fibronectin's III-1 module contains a conformation-dependent binding site for the amino-terminal region of fibronectin. J Biol Chem..

[18] Bazzoni G, Shih D-T, Buck CA, Hemler ME (1995). Monoclonal antibody 9EG7 defines a novel β1 integrin epitope induced by soluble ligand and manganese, but inhibited by calcium. J Biol Chem..

[19] Memmo LM, McKeown-Longo P (1998). The α_v_β_5_ integrin functions as an endocytic receptor for vitronectin. J Cell Sci..

[20] Busby TF, Argraves WS, Brew SA, Pechik I, Gilliland GL, Ingham KC (1995). Heparin binding by fibronectin module III-13 involves six discontinuous basic residues brought together to form a cationic cradle. J Biol Chem..

[21] Schwartz MA (1993). Signaling by integrins: implications for tumorigenesis. Cancer Res..

[22] Caccavari F, Valdembri D, Sandri C, Bussolino F, Serini G (2010). Integrin signaling and lung cancer. Cell Adh Migr..

[23] Gendron S, Couture J, Aoudjit F (2003). Integrin α2β1 inhibits Fas-mediated apoptosis in T lymphocytes by protein phosphatase 2A-dependent activation of the MAPK/ERK pathway. J Biol Chem..

[24] Albert JM, Cao C, Geng L, Leavitt L, Hallahan DE, Lu B (2006). Integrin αvβ3 antagonist Cilengitide enhnaces efficacy of radiotherapy in endothelial cell and non-small-cell lung cancer models. Int J Radiat Oncol Biol Phys..

[25] Zheng Y, Ritzenthaler JD, Roman J, Han S (2007). Nicotine stimulates human lung cancer cell growth by inducing fibronectin expression. Am J Respir Cell Mol Biol..

[26] Somanath PR, Kandel ES, Hay N, Byzova TV (2007). Akt1 signaling regulates integrin activation, matrix recogniton, and fibronectin assembly. J Biol Chem..

[27] Calleja V, Laguerre M, Parker PJ, Larigani B (2009). Role of novel PH-kinase domain interface in PKB/Akt regulation: structural mechanism for allosteric inhibition. PLoS Biol.

[28] Smith JW, Vestal DJ, Irwin SV, Burke TA, Cheresh DA (1990). Purification and functional characterization of integrin α_v_β_5_: an adhesion receptor for vitronectin. J Biol Chem..

[29] Han JY, Kim HS, Lee SH, Park WS, Lee JY, Yoo NJ (2003). Immunohistochemical expression of integrins and extracellular matrix proteins in non-small cell lung cancer: correlation with lymph node metastasis. Lung Cancer..

[30] Fornaro M, Plescia J, Chheang S, Tallini G, Zhu YM, King M, Altieri DC, Languino LR (2003). Fibronectin protects prostate cancer cells from tumor necrosis factor-α-induced apoptosis via the AKT/survivin pathway. J Biol Chem..

[31] Hetland G, Pettersen HB, Mollnes TE, Johnson E (1989). S-protein is synthesized by human monocytes and macrophages in vitro. Scand J Immunol..

[32] Salazar-Peláez LM, Abraham T, Herrera AM, Correa MA, Ortega JE, Paré PD, Seow CY (2015). Vitronectin expression in the airways of subjects with asthma and chronic obstructive pulmonary disease. PLoS ONE.

[33] Wang K, Seo BR, Fischbach C, Gourdon D. Fibronectin mechanobiology regulates tumorigenesis**.** Cell Mol Bioengin. 2015; doi: 10.1007/s12195-015-0417-4:1%E2%80%9311. 10.1007/s12195-015-0417-4 PMC4746220 26900407

[34] Morla A, Ruoslahti E (1992). A fibronectin self-assembly site involved in fibronectin matrix assembly: reconstruction in a synthetic peptide. J Cell Biol..

[35] Zhong C, Chrzanowska-Wodnicka M, Brown J, Shaub A, Belkin AM, Burridge K (1998). Rho-mediated contractility exposes a cryptic site in fibronectin and induces fibronectin matrix assembly. J Cell Biol..

[36] Hocking DC, Titus PA, Sumagin R, Sarelius IH (2008). Extracellular matrix fibronectin mechanically couples skeletal muscle contraction with local vasodilation. Circ Res..

[37] Yao R, Sui A, Wang Z, Liu S, Zhou Q, Liu X, Zhang H (2012). Induction of non-small cell lung carcinoma apoptosis using soluble RGD-TRAIL by targeting the integrin receptor of tumor cells. Mol Med Rep..

[38] Bordeleau F, Alcoser TA, Reinhart-King CA (2014). Physical biology in cancer. 5. The rocky road of metastasis: the role of cytoskeletal mechanics in cell migratory response to 3D matrix topography. Am J Physiol Cell Physiol.

[39] Kelsh RM, McKeown-Longo PJ (2013). Topographical changes in extracellular matrix: activation of TLR4 signaling and solid tumor progression. Trends in Cancer Res..

[40] Li B, Moshfegh C, Lin Z, Albuschies J, Vogel V (2013). Mesenchymal stem cells exploit extracellular matrix as mechanotransducer. Sci Rep.

[41] Kubow KE, Klotzsch E, Smith ML, Gourdon D, Little WC, Vogel V (2009). Crosslinking of cell-derived 3D scaffolds up-regulates the stretching and unfolding of new extracellular matrix assembled by reseeded cells. Integr Biol..

[42] Chandler EM, Saunders MP, Yoon CJ, Gourdon D, Fishbach C (2011). Adipose progenitor cells increase fibronectin matrix strain and unfolding in breast tumors. Phys Biol.

[43] Wan AM, Chandler EM, Madhavan M, Infanger DW, Ober CK, Gourdon D, Malliaras GG, Fischbach C (1830). Fibronectin conformation regulates the proangiogenic capability of tumor-associated adipogenic stromal cells. Biochim Biphys Acta..

[44] Wang X, Chen W, Zeng W, Bai L, Tesfaigzi Y, Belinsky SA, Lin Y (2008). Akt-mediated eminent expression of c-FGLIP and Mcl-1 confers acquired resistance to TRAIL-induced cytotoxcity to lung cancer cells. Mol Cancer Ther..

[45] Li Z, Xu X, Bai L, Chen W, Lin Y (2011). Epidermal growth factor receptor-mediated tissue transglutaminase overexpression couples acquired tumor necrosis factor-related apoptosis-inducing ligand resistance and migration through c-FLIP and MMP-9 proteins in lung cancer cells. J Biol Chem..

[46] Enyu L, Zhengchuan N, Jiayong W, Benjia L, Qi S, Ruixi Q, Cheng P, Khan AQ, Wei S, Jun N (2015). Integrin β6 can be translationally regulated by eukaryotic initiation factor 4E: contributing to colonic tumor malignancy. Tumour Biol..

[47] Byzova TV, Goldman CK, Pampori N, Thomas KA, Bett A, Shattil SJ, Plow EF (2000). A mechanism for modulation of cellular responses to VEGF: activation of the integrins. Mol Cell..

[48] Guidetti GF, Canobbio I, Torti M (2015). PI3K/Akt in platelet integrin signaling and implications in thrombosis. Adv Biol Regul..

[49] Aoudjit F, Vuori K (2012). Integrin signaling in cancer cell survival and chemoresistance. Chemotherapy Res Practice.

[50] Fedorenko IV, Abel EV, Koomen JM, Fang B, Wood ER, Chen YA, Fisher KJ, Iyengar S, Dahlman KB, Wargo JA et al. Fibronectin induction abrogates the BRAF inhibitor response of BRAF V600E/PTEN-null melanoma cells**.** Oncogene. 2015;15. doi:10.1038/-onc.2015.188. 10.1038/onc.2015.188 PMC4679729 26073081

[51] He Y, Wang Y, Liu H, Xu X, He S, Tang J, HGuang Y, Milao X, Wu Y, Wang Q (2015). Pyruvate kinase isoform (PKM2) participates in multiple myeloma cell proliferation, adhesion and chemoresistance. Leuk Res..

[52] Layani-Bazar A, Skornick I, Berrebi A, Pauker MH, Noy E, Silberman A, Albeck M, Longo DL, Kalechman Y, Sredni B (2014). Redox modulation of adjacent thiols in VLA-4 by AS101 convertys myeloid leukemia cells from a drug-resistant to drug-sensitive state. Cancer Res..

[53] Jiang X, Wang J, Zhang K, Tang S, Ren C, Chen Y (2015). The role of CD29-ILK-Akt signaling-mediated epithelial-mesenchymal transition of liver epithelial cells and chemoresistance and radioresistance in hepatocellular carcinoma cells. Med Oncol.

[54] Yi CH, Yuan J (2009). The Jekyll and Hyde functions of caspases. Dev Cell..

[55] Parrish AB, Freel CD, Kornbluth S. Cellular mechanisms controlling caspase activation and function**.** Cold Spring Harb Perspect Biol. 2013;5. doi:10.1101/cshperspect.a008672. 10.1101/cshperspect.a008672 PMC3660825 23732469

[56] Graf RP, Keller N, Barbero S, Stupack D (2014). Caspase-8 as a regulatory of tumor cell motility. Curr Mol Med..

[57] Graf R, Barbero S, Keller N, Chen L, Uryu S, Schlaepfer D, Stupack D (2013). Src-inducible association of CrkL with procaspase-8 promotes cell migration. Cell Adh Migr..

[58] Böger C, Kalthoff H, Goodman SL, Behrens H-M, Röcken C (2014). Integrins and their ligands are expressed in non-small cell lung cancer but not correlated with parameters of disease progression. Vichows Arch..

[59] Zheng M, Jones DM, Horzempa C, Prasad A, McKeown-Longo PM (2011). The first type III domain of fibronectin is associated with the expression of cytokines within the lung tumor microenvironment. J Cancer..

